# EGF-Induced Acetylation of Heterogeneous Nuclear Ribonucleoproteins Is Dependent on *KRAS* Mutational Status in Colorectal Cancer Cells

**DOI:** 10.1371/journal.pone.0130543

**Published:** 2015-06-25

**Authors:** Desamparados Roda, Josefa Castillo, Marcelino Telechea-Fernández, Anabel Gil, Gerardo López-Rodas, Luís Franco, Patricia González-Rodríguez, Susana Roselló, J. Alejandro Pérez-Fidalgo, Elena R. García-Trevijano, Andrés Cervantes, Rosa Zaragozá

**Affiliations:** 1 Department of Haematology and Medical Oncology, INCLIVA Biomedical Research Institute / University of Valencia, Valencia, Spain; 2 Department of Biochemistry and Molecular Biology, INCLIVA Biomedical Research Institute / University of Valencia, Valencia, Spain; University of Navarra School of Medicine and Center for Applied Medical Research (CIMA), SPAIN

## Abstract

*KRAS* mutational status is considered a negative predictive marker of the response to anti-EGFR therapies in colorectal cancer (CRC) patients. However, conflicting data exist regarding the variable response to EGFR-targeted therapy. The effects of oncogenic *KRAS* on downstream targets were studied in cell lines with different *KRAS* mutations. Cells harboring a single *KRAS^G13D^* allele showed the most tumorigenic profile, with constitutive activation of the downstream pathway, rendering them EGF-unresponsive. Conversely, *KRAS^A146T^* cells showed a full EGF-response in terms of signal transduction pathways, cell proliferation, migration or adhesion. Moreover, the global acetylome of CRC cells was also dependent on *KRAS* mutational status. Several hnRNP family members were identified within the 36 acetylated-proteins. Acetylation status is known to be involved in the modulation of EGF-response. In agreement with results presented herein, hnRNPA1 and L acetylation was induced in response to EGF in *KRAS^A146T^* cells, whereas acetyl-hnRNPA1 and L levels remained unchanged after growth factor treatment in *KRAS^G13D^* unresponsive cells. Our results showed that hnRNPs induced-acetylation is dependent on KRAS mutational status. Nevertheless hnRNPs acetylation might also be the point where different oncogenic pathways converge.

## Introduction

Colorectal cancer (CRC) is one of the most prevalent tumors worldwide [1] and despite many advances in therapy, long-term survival for patients with metastatic disease is still poor [2]. Antibodies against the Epidermal Growth Factor Receptor (EGFR) have been successfully used in CRC patients with advanced disease. However, less than half of them are responsive to such therapy [3]. *KRAS* or *NRAS* mutations are the main negative predictive markers to EGFR-response [4]. Therefore, treatment with anti-EGFR antibodies is only to be considered in patients with a full *RAS* wild-type phenotype [5, 6]. RAS proteins ensure signal transduction between membrane receptors, such as EGFR, and intra-cytoplasmic serine/threonine-kinases; thus contributing to the regulation of a number of essential cellular functions. Mutated RAS renders the protein into a constitutively active form, which in turn deregulates downstream signaling pathways [7]. However, several clinical and experimental data indicate that not all *RAS* mutations are equal in their biological properties and therefore, they could confer variable effects [8, 9]. The most frequent KRAS mutations found in CRC patients are in codon 12 and 13. However, activating *KRAS* mutations in codons 61 and 146 have been recently associated with shorter progression-free survival compared with wild-type *KRAS* in CRC-treated patients [10]. In addition, tumor cells under the pressure of inhibiting their oncogenic pathways develop spontaneous mutations. Indeed, metastatic CRC patients ongoing anti-tumoral treatment experience *KRAS* genotypic changes [11]. We also observed this effect in cultured cells; deletion of a mutated *KRAS* ^*G13D*^ allele in HCT116 cells (*KRAS* ^*G13D/WT*^) induced a spontaneous *KRAS* ^*A146T*^ mutation in the remaining wild type allele. To uncover the molecular mechanisms behind the differential response observed in tumor cells with different mutations in *KRAS* seems a major issue for development of new anti-tumoral therapies and personalized medicine.

Recently, a novel deacetylase-dependent mechanism has been proposed to explain resistance to anti-EGFR therapies in mutant *KRAS* lung adenocarcinoma cells [12]. Acetylation is a post-translational reversible modification regulated by two types of enzymes: lysine deacetylases (KDACs) and lysine acetyltransferases (KATs). Indeed, deacetylase inhibitors have emerged as potential anti-tumor agents by increasing hyperacetylation of both histones and nonhistone proteins [13]. Furthermore, some reports describe the interplay between KDAC inhibitors and the RAS-ERK signaling cascade in cell lines exhibiting different mutational status in *KRAS*, *BRAF or PI3KCA* [14–17].

The downstream effects of the less frequent, but not less important *KRAS* ^*A146T*^ mutation is currently unclear. In this article we evaluate the impact of *KRAS* ^*A146T*^ mutation *versus KRAS* ^*G13D*^ on cellular proliferation, adhesion and migration of HCT116-derived CRC cell lines. Given the recently described interplay between acetylations and RAS-ERK signaling cascades, we also studied the effect of KRAS mutational status on protein acetylation pattern in order to gain insight into the potential molecular mechanisms behind the differential effect of *KRAS* mutations.

## Material and Methods

### 2.1. Materials

Antibodies to hnRNPA1 (ab5832, ab50492), hnRNPA3 (ab50949), hnRNPA_2_/B_1_ (ab64800), hnRNPL (ab6106, ab65049) and GAPDH (ab8245) or β-actin (ab8227) as loading controls, were from Abcam. Antibodies against acetyl-Lys (9441), pAKT (40665) and AKT (9272) were from Cell Signaling Technology. Other antibodies used were: ERK (sc-93) and pERK (sc-7383) from Santa Cruz Biotechnology; KRAS (05–516) from Millipore and Talin (T3287) from Sigma-Aldrich.

Epidermal growth factor (EGF 20ng/ml), trichostatin (TSA, 0.5μM) and sodium butyrate were from Sigma-Aldrich, UO126 (10μM) from Promega, LY294002 (10μM) from Calbiochem and Fibronectin from BD Biosciences.

### 2.2. Cell culture

Colon cancer cell lines HCT116 and their derivatives HAE6 and HAF1 were commercially acquired from the GRCF Biorepository and Cell Center, at Johns Hopkins University. All cell lines were cultured in McCoy’s 5A Modified Medium (Gibco) supplemented with 10% FBS, penicillin/streptomycin and L-glutamine and maintained under standard conditions.

### 2.3. Protein extraction and Immunoblot analysis

Total proteins were extracted in RIPA buffer supplemented with inhibitors and sodium butyrate. Equal amounts of proteins were electrophoresed in SDS-PAGE gels and transferred onto nitrocellulose membranes and immunoblotted as described elsewhere [18]. Blots were developed by enhanced chemoluminiscence (ECL Detection Kit, GE Healthcare). Protein levels were quantified by densitometry and normalized by β-actin or GAPDH expression.

### 2.4. 2D-electrophoresis and acetylome analysis

Protein extracts (100μg) were separated by two-dimensional (2D) electrophoresis, as previously described [18]. Proteomic images were acquired with a Typhoon Trio Imager. In parallel, 2D-gels were electroblotted on nitrocellulose membranes and analyzed by western blot with anti-acetylated lysine antibody. Acetylated proteins overlapping with the SYPRO Ruby-stained gels were excised for MS analysis. Gel specimens were processed with a MassPrep station (Waters). NanoLC-ESI-MS/MS and data analysis were performed as described [19] by the Proteomics and Bioinformatics Unit, (CIMA-University of Navarra, Spain).

### 2.5. Immunoprecipitation

500μg of proteins were immunoprecipitated overnight with specific antibodies or normal serum IgG (Dako) at 4°C on a rotating device. Protein-antibody complexes were pulled-down with 50% (v/w) Dynabeads (Invitrogen, Life Technologies). After several washes in PBS, immunocomplexes were recovered by boiling in LB and then analyzed by Western blot as described. 1% of protein extracts used for immunoprecipitation was loaded as Input.

### 2.6. RNA isolation and expression analysis by qPCR

RNA extraction, complementary DNA synthesis and qRT-PCR were carried out as preciously described. [18]. Primer sequences for *hnRNPA1*, *hnRNPA3*, *hnRNPA2/B1*, *hnRNPL* and *GAPDH* are shown in supplementary data. Pre-developed Taqman primers for *KRAS*, *ADAM19*, *CATHEPSIN C*, *CATHEPSIN L* and *18S* were from Applied Biosystems. Relative gene expression was expressed as 2^-Δ(ΔCt)^ Data were normalized by *18S* quantification in the same sample reaction. All reactions were carried out in triplicate.

### 2.7. Microarray gene expression. Hybridization and data analysis

Total RNA (5μg) was used to get biotinylated cRNA according to the manufacturer's protocol (Affymetrix). Quality of the labelled cRNA was confirmed by hybridization to an Affymetrix test chip (Test3-chip). Labelled cRNA was hybridized to an Affymetrix Genechip Human Gene 1.0ST. Microarray data were obtained by AGCC Affymetrix software. Raw data of three replicates for each cell line were analyzed by R/Bioconductor using the Limma package [20]. F-statistics, t-statistics and log odds of differential expression were calculated by empirical Bayes moderation of the standard errors towards a common value. Differentially expressed genes were identified by pairwise comparison, (p-value cutoff = 0.05). Only genes with a fold change above 1.5 and below -1, were considered differentially expressed for further analyses and classified according to the biological process category following the criteria of the Gene Ontology Consortium [21].

### 2.8. BrdU Cell proliferation assay

Cell proliferation was examined by a bromodeoxyuridine (BrdU) incorporation assay kit (Calbiochem). Briefly, the cells were seeded at a density of 5x103 cells/ well in a 96-well culture plate. After 24h starvation, cells were treated with EGF (20 ng/ml) in serum-free medium. The BrdU reagent was added to the wells for 4h in the presence or absence of EGF. At the indicated time points, cells were fixed during 30min in a fixative/denaturing solution. Cells were incubated with anti-BrdU antibody (1:100) for 1h and after several washes, incubated with HRP-conjugated IgG for 30 min. Tetra-methyl benzidine substrate was then added for 15 min and the reaction was stopped by the addition of 1.25M sulfuric acid stop solution. Absorbance was measured in each well at dual wavelengths 450–540nm.

### 2.9. Wound-healing assay

The three cell lines were cultured under standard conditions. Confluent monolayers were scratched with a 10μl pipette tip to induce a wound. The wounded edges were imaged using an inverted microscope Nikon Eclipse Ti (10X Magnification). Images were collected at 0, 24 and 48 h after scratch.

### 2.10. Statistical analysis

Data are the mean ± SEM of at least three independent experiments. Significant differences were determined by one-sample Student’s t test. Differences were considered significant at *p*<0.05. Representative blots are shown.

## Results

### *3*.*1*. Differential effect of *KRAS* ^*A146T*^ *and KRAS* ^G13D^ in EGF-treated CRC cells

HCT116 CRC cell line, harboring an endogenous activating *KRAS* ^G13D/wt^ mutation, and two isogenic HAF1 (*KRAS* ^wt/-^) and HAE6 (*KRAS* ^G13D/-^) clones were used for this study. All those genes recommended to predict a clinical outcome for cancer therapy were sequenced in the three cell lines [22] (S1 Table). Interestingly, while all mutations found in HCT116 were also observed in HAE6 cells, we found that in HAF1 cells a *KRAS* ^*A146T*^ hotspot mutation had spontaneously arisen in what so far was intended to be a wild-type allele. Given this result, we characterized *KRAS* ^*A146T*^ cells and compared them with HCT116 or HAE6 cell lines (S1 Fig and S1 File). HAE6 cells showed the highest proliferation rate and the lowest levels of cell adhesion. A wound healing analysis of cell migration revealed differences among the three cell lines (, with HCT116 showing a higher rate than HAF1 but lower than HAE6 cells. In contrast to the previous results, *KRAS* mutational status had no apparent effect on ERK and AKT phosphorylation, under basal conditions. This is not surprising, since the three cell lines harbor a PIK3CA activating mutation.

**Fig 1:**
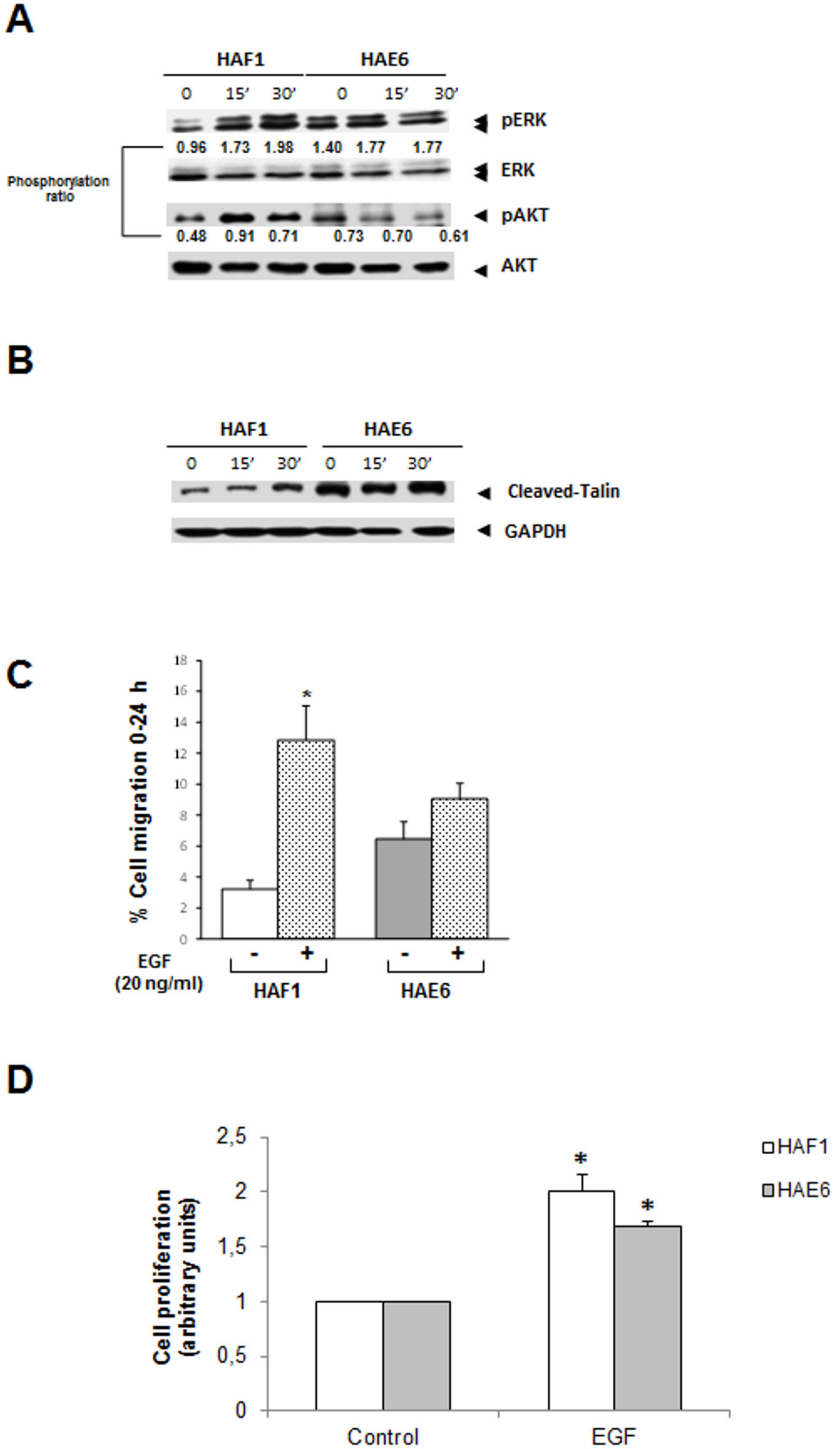
KRAS downstream pathway, adhesion and migration in EGF-treated cells. **A**. Downstream targets of KRAS signaling pathway were analyzed by western blot to determine pAKT and pERK1/2 levels after EGF-treatment. **B**. Cell adhesion was determined by western blot analysis of TALIN cleavage in HAF1 and HAE6 cells. **C**. Migration ratio was studied after EGF (20ng/mL) treatment by wound healing assay. Graph shows the percentage of cells that migrated to the wound area after 24h. **D**. Cell proliferation, measured by BrdU assay, to analyze proliferative activity in EGF-treated (20ng/mL) cells. *P<0.05 EGF-treated vs untreated controls (n = 4).

*KRAS* ^*A146T*^ seemed to be the less tumorigenic mutation. Nevertheless, unsynchronized cell culture under basal conditions could mask the effect of KRAS mutation in response to a specific stimulus. Therefore, to further explore the downstream effect of *KRAS* ^*A146T*^ versus the well-characterized *KRAS* ^*G13D*^ mutation, HAF1 and HAE6 cells were grown in serum-deprived media and then stimulated with EGF. To rule out the possibility that the mutation could affect *KRAS* expression, qPCR was performed and *KRAS* mRNA levels found in both cell lines were similar (data not shown). Although EGF-treatment induced the rapid phosphorylation of ERK1/2 and AKT in HAF1, this response was poor in HAE6 cells (Fig 1A). Noticeable, pERK levels were already high in untreated HAE6 controls. In addition, as already described in other cell lines with an hyperactivated ERK pathway [23], EGF decreased pAKT levels in this HAE6 cell line.

Cell adhesion experiments could not be performed in serum-free media because HAF1 cells were sensitive to trypsinization after 24h-serum deprivation. A higher talin-cleavage has been associated with lower cell adhesion [24]. Therefore, we analyzed cleaved-talin as an indirect method to measure cell adhesion. In unstimulated cells, talin-cleavage was already higher in HAE6 than in HAF1 cells. Moreover, while talin-cleavage was induced in response to EGF in HAF1 cells, this proteolytic cleavage was EGF-independent in HAE6 cells (Fig 1B). We next analyzed cell migration and found that HAE6 cells were also unresponsive to EGF, in contrast to the cell migration induced by EGF in HAF1 cell line (Fig 1C). Finally, cell proliferation, although induced by EGF in both cell lines, was increased to a lesser extent in HAE6 than in HAF1 cells (Fig 1D). We reasoned that hyperactivation of ERK1/2 induced by KRAS^G13D^ could reach a plateau beyond which cells cannot be further stimulated by EGF.

### 3.2. Differential mRNA levels in KRAS^G13D^ vs KRAS^A146T^ CRC cell lines

**Fig 2:**
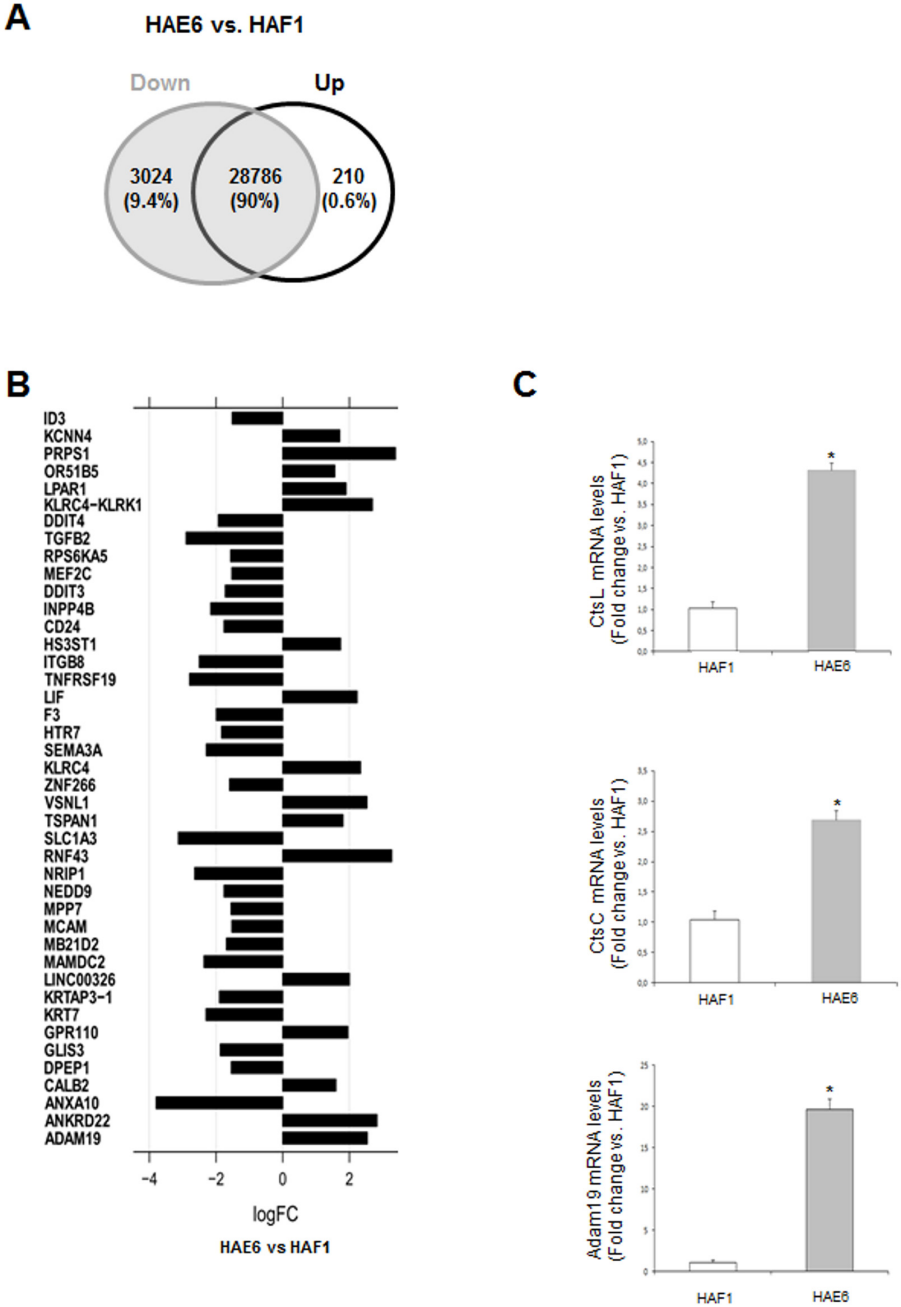
Gene expression profile from Microarray data analysis. Total RNA from CRC cells cultured in basal conditions with 10% FBS was analyzed by microarray. **A**. Venn-diagram showing the percentage of up- and down-regulated genes found in the cell line harboring a *KRAS* ^*G13D*^ mutation (HAE6), when compared to mRNA levels found in HAF1 cells (*KRAS* ^*A146T*^ *)*. **B**. Bar chart representing differentially expressed genes (p-value ≤ 0.001) among the two cell lines HAE6 *versus* HAF1 cells, considering only-log2 fold change >1.5 and <-1.5. Gene symbol for each gene is indicated on the left. **C**. Three genes, *CATHEPSIN L*, *CATHEPSIN C* and *ADAM19* were selected based on their potential role on cell invasion and migration, for microarray validation using qPCR. *P< 0.05 versus HAF1 cells.

We examined those possible KRAS-dependent transcriptional effects in both cell lines using Affymetrix DNA microarrays (S2 and S3 Files). Under the presence of *KRAS* ^*G13D*^ mutation, most differentially expressed genes were down-regulated (Fig 2A and 2B; S2 Fig) when compared to *KRAS* ^***A146T***^ mutation (HAF1). However, the expression of the majority of genes did not substantially change, and only few of them were up-regulated. In order to validate these data, the expression of a selection of up-regulated genes was analyzed by qPCR. These genes were selected for their prominent role in tumor metastasis, growth factor shedding or CRC drug-resistance [25, 26]. The expression of these genes was dramatically up-regulated in cells with a mutated *KRAS* ^*G13D*^ allele, thus confirming the microarray data (Fig 2C and S2 Fig). Among the genes down-regulated in HAE6 vs. HAF1 cells, the most representative pathway affected was EGFR1 signaling; 43 genes were down-regulated out of the 156 described for the EGFR1 canonical pathway. However, other pathways triggered by cytokines and growth factors, such as VEGF, PDGF, HGF, IGF1,TGFβ, TNFα and several interleukins, were also down-regulated. This is not surprising since most of them share several steps within the RAS signaling pathway.

### 3.3. Effect of KRAS mutations on the global acetylome of CRC

**Fig 3:**
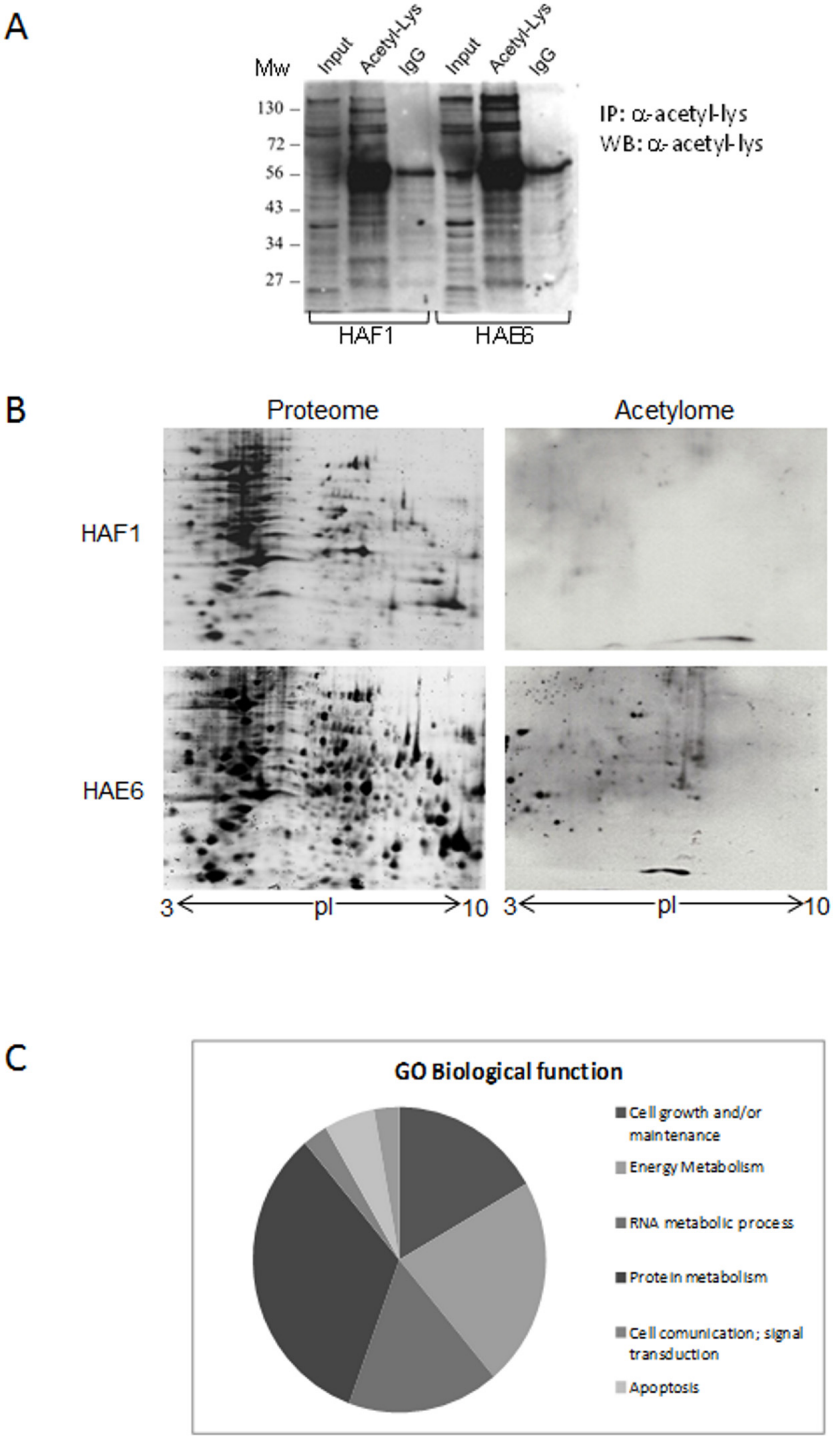
Acetylome analysis in CRC cell lines. **A**. Cell homogenates were immunoprecipitated with anti-acetyl-lysine antibody and the precipitates were then immunoblotted against acetyl-lysine residues, including an equal amount of starting homogenate (named Input). IgG: Homogenates immunoprecipitated with normal serum IgG. Molecular mass markers (kDa) are on the left of the gel. **B**. Protein expression profiles of both cell lines separated by IEF two-dimensional PAGE, stained with SYPRO Ruby (left panel) or immunoblotted against acetyl-lysine antibody to identify acetylated proteins (right panel). The pH increases from left to right on the abscissa and the molecular mass decreases from top to bottom on the ordinate. **C**. Distribution of GO Biological functions assigned to the acetylated proteins identified in the acetylome of HAE6 cells.

One of the potential pathways affected by KRAS mutational status which may regulate several biological processes is protein acetylation. We sought to study whether the global acetylation profile in CRC was differentially affected by activated KRAS^G13D^ and activated KRAS^A146T^. Isolated proteins from both cell lines grown in 10%FBS, were immunoprecipitated and analyzed by western blot with anti-acetyl-Lys antibodies (Fig 3A). A 2D western blot proteomic approach was used to deeply analyze acetylated-proteins from the two cell lines. Global protein Lys-acetylation was increased in HAE6 compared to HAF1 cells, according to increased number of detected spots (Fig 3B).

Positive spots from the HAE6 acetylome were excised and identified by mass spectrometry. 67 spots were detected but only those with a confident Mascot ion score and a high peptide matching were selected (S2 Table). The (GO) biological function analysis of our 36 acetylated proteins revealed that target proteins were enriched in four main categories related to cell growth, energy metabolism, RNA metabolic process and protein metabolism (Fig 3C). Many of these proteins were already described to be targets of putative acetyltransferases or deacetylases and to become acetylated under different experimental conditions (reviewed in S2 Table). Our microarray results confirmed that differences in the acetylation pattern were not due to increased mRNA expression. Indeed, those genes whose products were identified in our acetylome analysis remained unchanged in HAE6 when compared to HAF1 cells (S2 and S3 Files).

### 3.4. Acetylation of hnRNP family members

**Fig 4:**
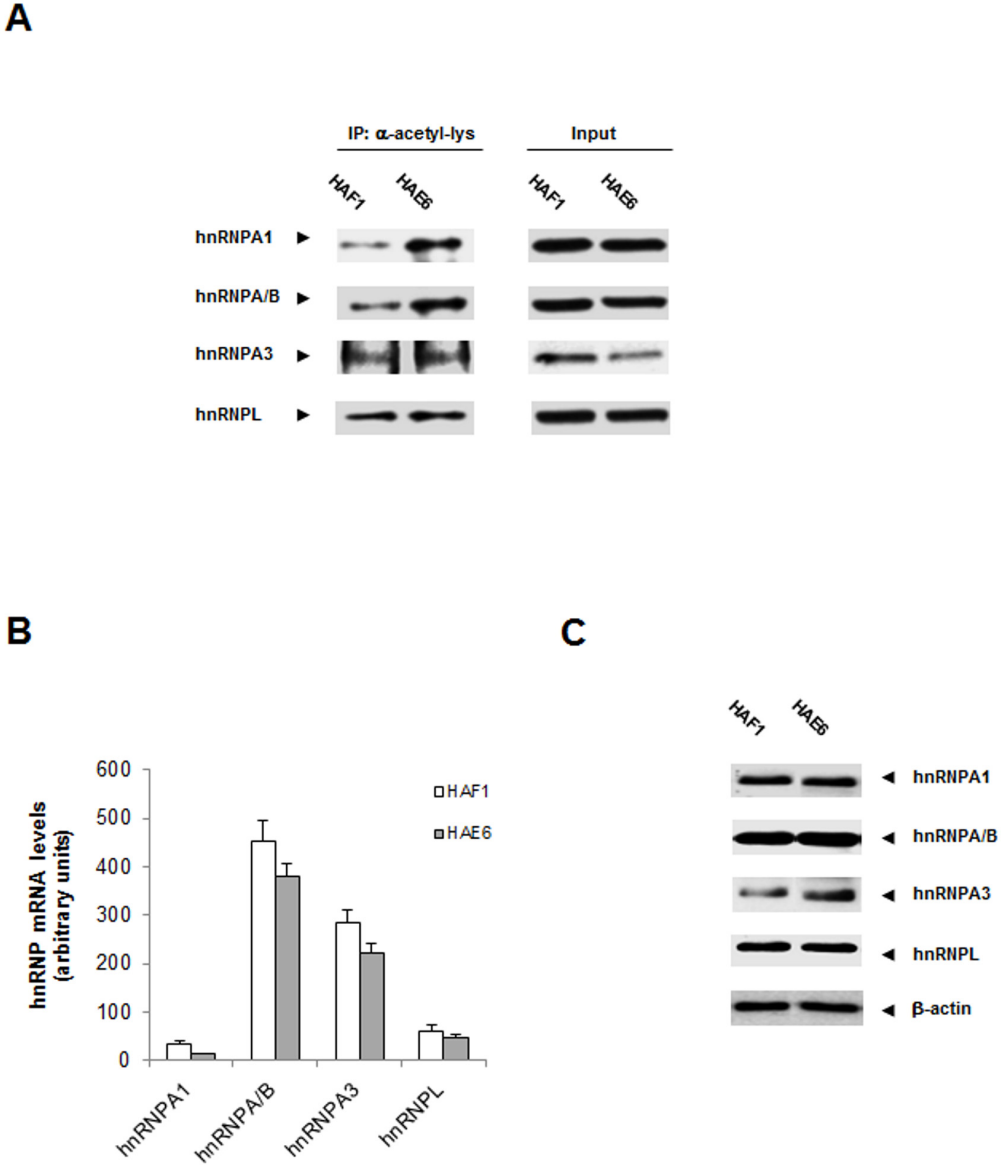
hnRNPs acetylation in CRC *KRAS* ^*G13D*^ and *KRAS* ^*A146T*^ cells. **A**. Total extracts from the two CRC cell lines under basal conditions (10% FBS) were isolated and immunoprecipitated with α-acetyl-lysine antibodies. The immunoprecipitated sample was then analyzed by western blot with antibodies recognizing the specific hnRNP member (left panel). Inputs of each specific hnRNP in the different cell lines are shown (right panel). **B**. mRNA levels of hnRNPs were analyzed by qPCR (n = 3). No statistical significance was found. **C**. Western blot analysis showing protein levels of the different hnRNPs in HAF1 and HAE6 cells. β-actin was used as loading control.

Among the acetylated-targets there was an interesting group of RNA binding proteins, all of them members of the family of heterogeneous nuclear ribonucleoproteins (hnRNPs), involved in a variety of molecular functions going from mRNA processing, transport, stability or even translation [27]. We analyzed in the two cell lines whether the basal acetylation of hnRNPs was dependent on *KRAS* mutational status. Acetylation was measured by immunoprecipitation experiments with acetyl(Lys) antibodies followed by western blot with antibodies recognizing the specific hnRNP member. As shown in Fig 4A, hnRNPA1 and A2/B1 were found to be hyperacetylated under growing conditions (10% FBS) in HAE6 when compared to HAF1; however, this pattern was not so clear in other family members tested such as hnRNPA3 or L. The mRNA and protein levels of hnRNPs were analyzed by qPCR and western blot respectively (Fig 4B and 4C); the expression of none of the hnRNPs showed a significant change when compared among the different cell lines. Therefore, the different levels of acetylation observed are not the result of a higher rate of gene expression or protein stabilization.

### 3.5. Acetylation of hnRNPA1 and L in response to EGF

**Fig 5:**
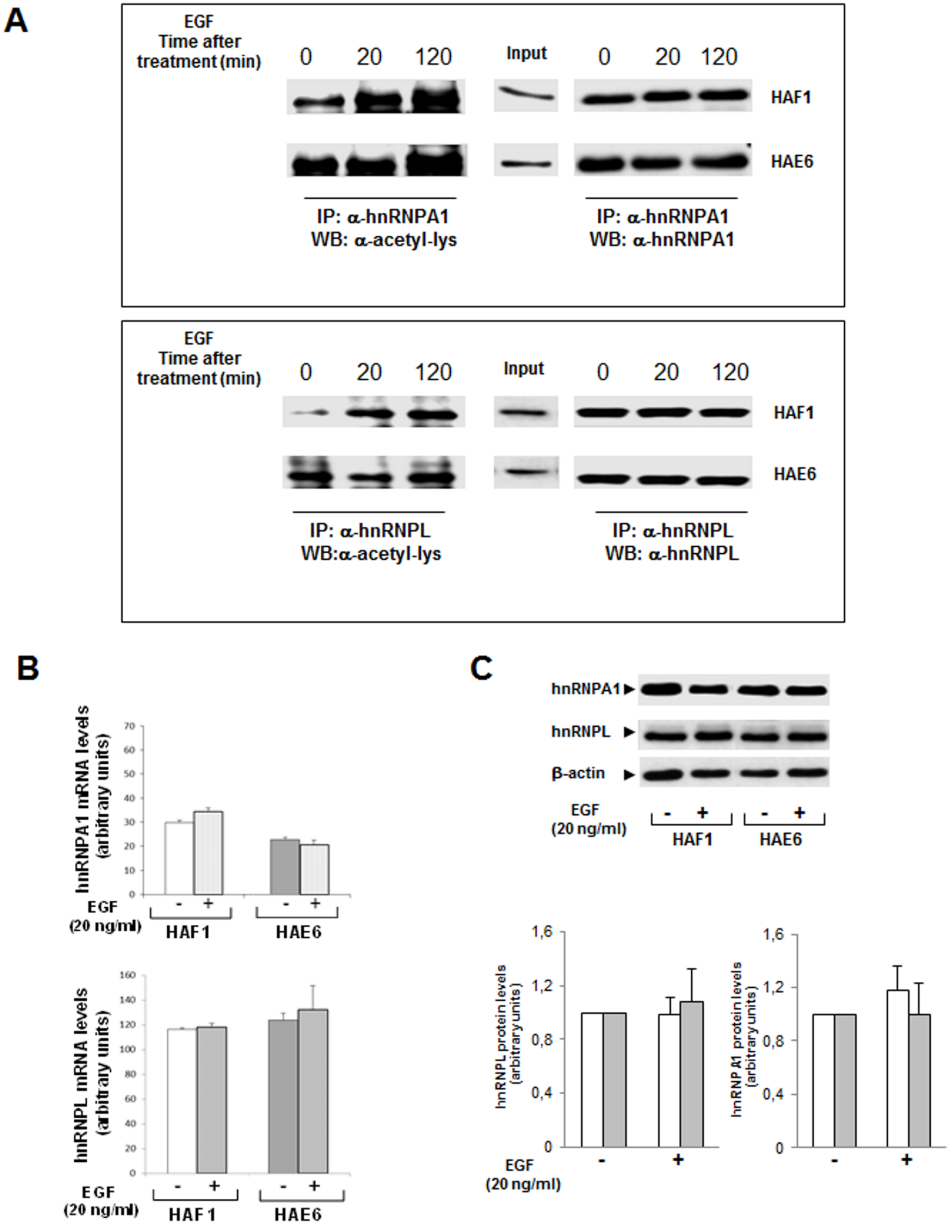
Effect of EGF treatment on hnRNPs acetylation in *KRAS* mutated cells. Both CRC cell lines were grown in serum-free medium and then stimulated with EGF (20ng/mL) for 20 and 120min. **A**. Protein extracts from control or EGF-treated cells were isolated and immunoprecipitated with α-hnRNPA1 (upper panel) or hnRNPL (lower panel) antibodies. The immunoprecipitated samples were then analyzed by western blot with antibodies recognizing acetyl-lysine residues and, either hnRNPA1 or hnRNPL. Inputs of each specific hnRNP in the different cell lines are shown. **B**. mRNA levels of hnRNPA1 and hnRNPL were analyzed by qPCR in control and 2h EGF-treated cells. No statistical significance was found (n = 3). **C**. Western blot analysis showing protein levels of both hnRNPs in HAF1 and HAE6 cells in control and 2h EGF-treatment conditions. The intensity of hnRNPs bands was measured and normalized by β-actin; graphs in lower panel show the quantification for both hnRNPL and A1 in HAF1 (white bars) and HAE6 (grey bars) cell lines.

Although acetylation of hnRNPs has been already described in other cell systems, at present it is not known whether this acetylation is part of the EGF response in CRC cells. Should it be the case, it will be important to establish whether the *KRAS* mutational status could condition a differentially induced-acetylation of hnRNPs upon EGF stimuli. Both cell lines were grown in serum-free medium and then stimulated with EGF for a short (20min.) and a long (2h) period of time (Fig 5A). Samples from each condition were immunoprecipitated with either anti-hnRNPA1 or anti-hnRNPL antibodies. These hnRNPs were selected since they represent those isoform showing the highest and the lowest acetylation differences when comparing HAF1 and HAE6 cells. To establish the acetylation ratio (acetyl-hnRNP/total hnRNP), pull-down proteins were further analyzed by western blot with specific antibodies against acetyl-Lys or the corresponding hnRNP.

Under serum deprivation, basal acetylation levels of both hnRNPs were higher in HAE6 than in HAF1 cells (Fig 5A, lane 0). However, the acetylation of both, hnRNPA1 and hnRNPL at the end point of EGF-treatment was the same in the two cell lines. Interestingly, when the acetylation of EGF-treated samples was compared to untreated controls, acetylated-hnRNP levels did not significantly change throughout the time course of EGF-treatment in HAE6 cells (Fig 5A). Conversely, EGF induced a dramatic acetylation of both hnRNPs in HAF1 cells. Interestingly the same response was observed after EGF treatment in HCT116 parental cell line (S3 Fig). Finally, mRNA and protein levels from hnRNPA1 or-L were not induced in any EGF-treated cell line at the tested time points (Fig 5B and 5C; S3 Fig). Consequently, EGF-induced acetylation observed in HAF1 cannot be attributed to the transcriptional or translational modulation of hnRNPs.

### 3.6. Role of *KRAS* ^A146T^ mutation on hnRNP acetylation

**Fig 6:**
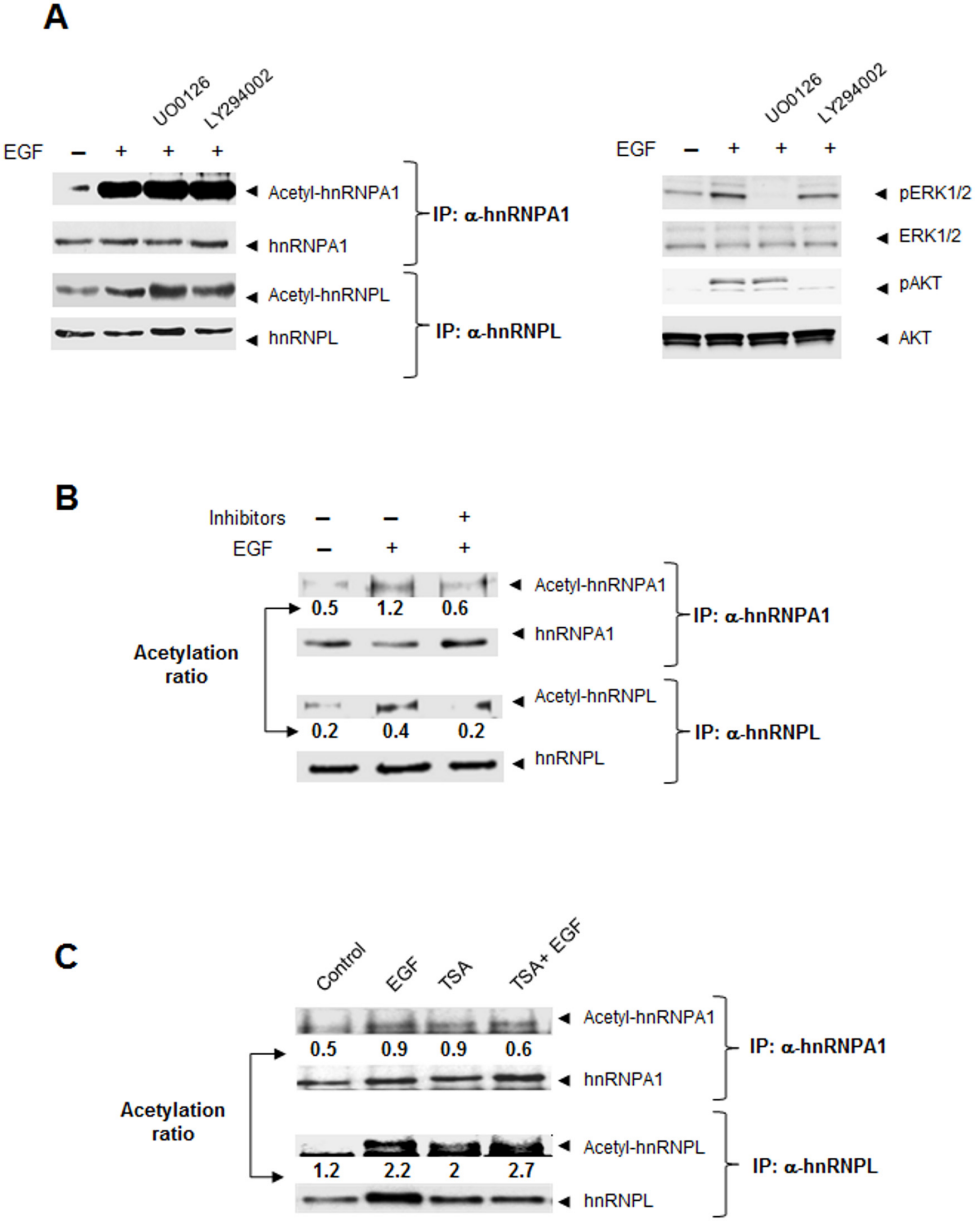
Role of KRAS^A146T^ mutation on downstream pathway and hnRNP acetylation. **A**. HAF1 cells were treated with EGF in the presence or absence of MAPK inhibitors (MEK, UO0126 or PI3KA, LY294002). Protein extracts from HAF1 cells were immunoprecipitated with α-hnRNPA1 or hnRNPL antibodies. The immunoprecipitated samples were then analyzed by western blot with antibodies recognizing acetyl-lysine residues and, either hnRNPA1 or hnRNPL (Left panel). Phosphorylation of ERK1/2 and AKT in HAF1 cells was also assessed to confirm efficiency of individual inhibitors at doses used (Right panel). **B**. Both MAPK inhibitors were simultaneously used to study their effect on EGF-induced acetylation of hnRNPs. **C**. Role of deacetylases on basal acetylation levels of hnRNPs were further studied in HAF1 cells treated with trichostatin A. Immunoprecipitation with hnRNPs antibodies and western blot against acetyl-lysine, hnRNPA1 or L in HAF1 cells treated with EGF, TSA or a combination of both are shown.

To study whether *KRAS* ^*A146T*^ mutation may account for EGF-induced acetylation of hnRNPs, we specifically inhibited the KRAS signaling pathway in the HAF1 cell line. Independently of *KRAS* ^A146T^ mutation, HAF1 cells also encompass a *PI3KCA* mutation. Therefore we analyzed the acetylation of hnRNPs induced by EGF in cells pretreated with inhibitors of both, MEK1/2 (U0126) and PI3K (LY294002). Inhibition of one single pathway either MEK1/2 or PI3K, by using the specific inhibitor alone, had no effect on protein acetylation (Fig 6A). The ability of RAS to influence PI3K activity and vice versa has been well documented [28, 29]. Indeed, blocking MEK1/2 activity has been shown to induce AKT phosphorylation in CRC cell lines [30]. On the other hand, blocking PI3K activity has been shown to increase pERK1/2 levels in response to a variety of stimulus [28]. In agreement with this, when both inhibitors were used simultaneously, the inhibition of RAS/PI3K signaling pathways completely blocked the EGF-induced acetylation of both hnRNPA1 and hnRNPL (Fig 6B). Interestingly, basal levels of acetylated hnRNPs in serum-starved controls were not affected by the kinases inhibitors.

Basal levels of acetyl-hnRNPs could be dependent on the half-life of acetylations. Actually, acetylation of proteins is not only the result of acetyltransferase-induced activity, but also of deacetylases. To examine the role of deacetylases on basal acetylation, we investigated whether the deacetylase inhibitor trichostatin A (TSA) could mimic the effect of EGF in HAF1 cells. Indeed, TSA increased the acetylation of both hnRNPs to the same levels as those reached by the EGF-treatment, although no synergistic effect was observed (Fig 6C).

## Discussion

Herein we uncover several aspects of the molecular events underlying colon carcinogenesis that will be further discussed. We provide the first report indicating that the acetylation of 36 specific proteins is dependent on *KRAS* mutational status; among them, several members of the hnRNP family. Moreover, our data indicate that hnRNP acetylation is part of the EGF-inducible response. Secondly, we show the molecular consequences of allele imbalance by deletion of either, a wild-type or a mutant allele in *KRAS* ^*G13D/WT*^ CRC cells. Deletion of the wild type allele (HAE6) leads, not only to hnRNP acetylation, but also to the more tumorigenic and EGF-unresponsive profile of these cells. Conversely, deletion of the mutant allele (HAF1) induces a spontaneous *KRAS* ^*A146T*^ mutation in the wild-type allele.

Collectively our observations seem to parallel the clinical findings indicating that disruption of the *KRAS* wild-type allele is an adverse prognostic factor for CRC [31]. Surprisingly, in HAF1 cells, a spontaneous *KRAS* ^*A146T*^ point mutation was found. In agreement with this, the most recent reports show dynamic *KRAS* genotypic changes throughout the progression of metastatic CRC [11]. This point mutation also renders a constitutive activation of KRAS [32], In the near future, it would be important to analyze possible spontaneous mutations in any other CRC cell line used as experimental model to study the molecular mechanisms of therapy-resistance or new drug development.

Mutation screening tests based on the hotspot *KRAS* codons 12 and 13 have resulted in the mis-classification of up to one-third of patients that were erroneously considered as *KRAS* wild-type and were resistant to anti-EGFR treatments [33, 34]. *KRAS* mutations in codons 61 and 146 are significantly associated with shorter progression-free survival compared with wild-type *KRAS* in CRC patients treated with a combination of cetuximab and chemotherapy [10]. Although many questions remain to be answered to uncover the impact of *KRAS* ^*A146T*^ mutation in CRC drug resistance, to our knowledge this is the first description of the effect of this recently discovered mutation on downstream signaling pathways, proliferation, migration and cell adhesion in EGF-stimulated CRC cells. Thus, our results further reinforce the importance of extending the mutation screening tests to the less frequent hotspot mutations such as codon 146 or 61 [5, 22], which could determine the EGF-response going from signal transduction to the global acetylome of tumor cells.

Indeed, the acetylation/deacetylation process is involved in the modulation of the EGF-response in experimental and clinical CRC studies [15]. However, how the mutational status of *KRAS* can affect the acetylome of CRC is so far not known. In agreement with our observations about hnRNPs acetylation, one of the largest acetylome studies showed that the main functional networks modulated by acetylation were related to RNA maturation [35]. All in all, although the prominent role of hnRNPs in tumor progression has been already described [36–38], this is the first report showing a relation between *KRAS* mutational status and hnRNP acetylation. The highest level of acetyl-hnRNPs, and also the largest number of acetylated proteins, was found in cells harboring one single allele of *KRAS* ^*G13D*^. Accordingly, our molecular and biological characterization of *KRAS* ^*G13D*^ cell line confirmed the more oncogenic behavior of these cells (HAE6).

Indeed, under basal conditions, while proliferation and migration rates were dramatically higher, cell adhesion was lower in HAE6 (*KRAS* ^*G13D/-*^) than in HAF1 cells (*KRAS* ^*A146T/-*^). It has been proposed that oncogenic KRAS might promote tumor progression by limiting the efficacy of RAS/RAF/MEK/ERK1/2 signaling, whereas KRAS—responsive tissues exhibit a full activated signaling pathway, and trigger potent antitumor responses [31]. In agreement with this, HAE6 cells showed an EGF-unresponsive MAPK signaling pathway. Consistently, the acetylation of hnRNPL and A1 remained unchanged after EGF-treatment. Furthermore, EGF could not further induce in these cells, any of the biological responses described for this growth factor, i.e. cell proliferation, adhesion or migration.

The functional consequences of hnRNP acetylation are still unknown; however, in agreement with a KRAS-dependent modulation of hnRNP acetylation, several hnRNP family members are modulated by the MAPK pathway [39, 40]. This phosphorylation may condition hnRNP subcellular distribution and function [28]. The possible interplay between acetylation and phosphorylation of specific hnRNPs remains elusive. In the near future, it would be important to study whether this putative interplay could determine mRNA splicing, stability, transport or translation of specific genes involved in tumorigenesis or resistance to anti-tumor therapies. On the other hand, since CRC cells also harbor KRAS-independent mutations, we cannot rule out the suggestive idea of hnRNPs acetylation as the point where different signaling pathways converge. The mutation-induced activation of one or more of these pathways might result in hnRNPs hyperacetylation. Should this hypothesis be confirmed, the relevance and extension of our data will be increased from both, the molecular and clinical point of view.

## Supporting Information

S1 Fig Proliferation, adhesion and migration of CRC isogenic cells in basal conditions. **A**. HAE6, harboring a single *KRAS* ^G13D^ mutated allele, showed increased proliferation compared to HAF1 and HCT116 isogenic cell lines, as determined by MTT cell proliferation assays. A viability assay was conducted daily from 1 to 4 days. The results are expressed as fold *versus* day 1 for each cell line. **B**. Effect of *KRAS* ^G13D^ mutation on cell adhesion in fibronectin-coated plates (2,5 μg/cm^2^). The results are expressed as fold compared to HAF1 cell line. **C**. Wound-healing assay was performed to analyze cell migration. The micrographs show the wound (left panels), cell migration at 24h (middle panels) and at 48h (right panels) for the three cell lines. Tumor cells, which migrated to the wound area, were counted at 48h. The results are expressed as fold compared to HAF1 cell line. **D**. Phosphorylated and total ERK1/2 protein levels, as well as AKT and pAKT levels were analyzed by western blot in the three cell lines cultured in basal conditions with 10%FBS. Image shown is representative of three independent experiments. Results are means ± S.E.M. of four independent experiments. *P< 0.005 versus HAF1 cells; ^#^P<0.005 versus HCT116 cells.
(TIF)

S2 Fig HCT116 Gene expression profile from Microarray data analysis. Total RNA from HCT116 CRC cells cultured in basal conditions with 10% FBS was analyzed by microarray. **A**. Venn-diagram showing the percentage of up- and down-regulated genes found in the parental cell line harboring a *KRAS* ^*G13D*^ mutation (HCT116), when compared to mRNA levels found in HAF1 cells (*KRAS* ^*A146T*^ *)*. **B**. *CATHEPSIN L*, *CATHEPSIN C* and *ADAM19* were compared using qPCR. *P< 0.05 versus HAF1 cells.
(TIF)

S3 Fig EGF-induced acetylation of both hnRNPA1 and hnRNPL in HCT116 cells. HCT116 cells were grown in serum-free medium and then stimulated with EGF (20ng/mL) for 20 and 120min. **A**. Protein extracts from control or EGF-treated cells were isolated and immunoprecipitated with α-hnRNPA1 or hnRNPL antibodies. The immunoprecipitated samples were then analyzed by western blot with antibodies recognizing acetyl-lysine residues and, either hnRNPA1 or hnRNPL. Inputs of each specific hnRNP in the different cell lines are shown. **B**. mRNA levels of hnRNPA1 and hnRNPL were analyzed by qPCR in control and 2h EGF-treated cells. No statistical significance was found (n = 3). **C**. Western blot analysis showing protein levels of both hnRNPs in control and 2h EGF-treatment conditions. The intensity of hnRNPs bands was measured and normalized by β-actin; graph shows the quantification for both hnRNPL and A1 in HCT116 cell line. No statistical significance was found (n = 3).
(TIF)

S1 File Supplementary Material and Methods: Cell viability, Short term cell adhesion and Somatic mutations sequencing. (DOCX)

S2 File Microarray raw data of HAF1 and HAE6 cell lines under basal conditions (10% FBS). (XLS)

S3 File Normalized Comparison of Microarray data from HAF1 and HAE6 cell lines under basal conditions (10% FBS). (XLSX)

S1 Table Genes and codons sequenced for detection of somatic mutations in CRC cell lines. (DOCX)

S2 Table Acetylated proteins identified in HAE6 cells and reported KATs/ KDACs interactions. (XLSX)
