# Supplementary figures and images for: EGF-Induced Acetylation of Heterogeneous Nuclear Ribonucleoproteins Is Dependent on KRAS Mutational Status in Colorectal Cancer Cells
Source: PLoS One. 2015 Jun 25;10(6):e0130543. doi: 10.1371/journal.pone.0130543 (PMC4482484; doi:10.1371/journal.pone.0130543)

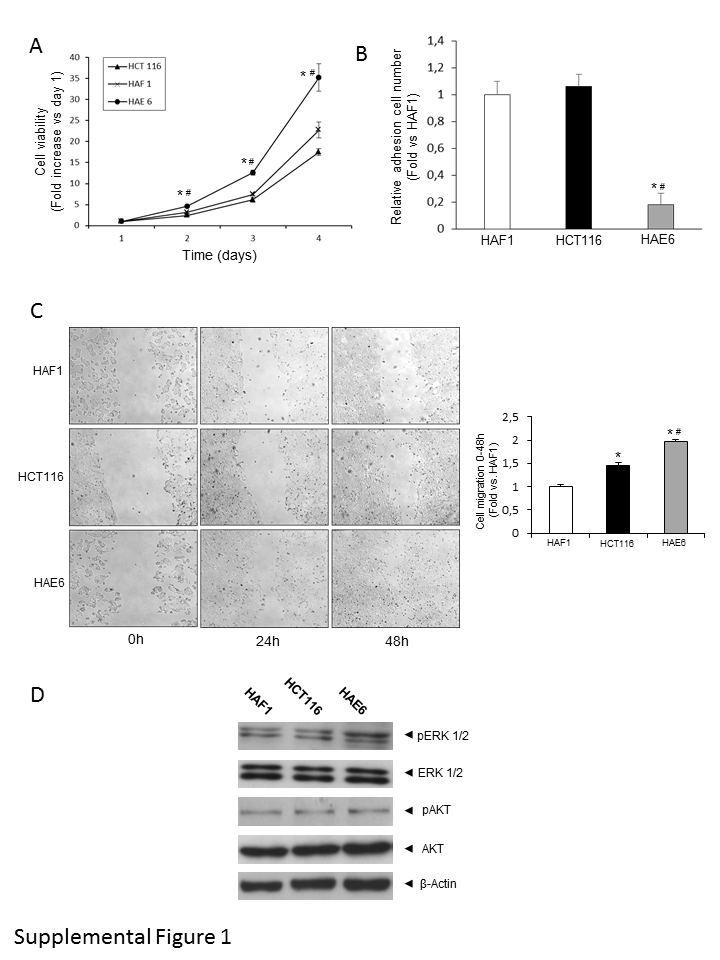

Supplement: S1 Fig — A. HAE6, harboring a single KRAS G13D mutated allele, showed increased proliferation compared to HAF1 and HCT116 isogenic cell lines, as determined by MTT cell proliferation assays. A viability assay was conducted daily from 1 to 4 days. The results are expressed as fold versus day 1 for each cell line. B. Effect of KRAS G13D mutation on cell adhesion in fibronectin-coated plates (2,5 μg/cm2). The results are expressed as fold compared to HAF1 cell line. C. Wound-healing assay was performed to analyze cell migration. The micrographs show the wound (left panels), cell migration at 24h (middle panels) and at 48h (right panels) for the three cell lines. Tumor cells, which migrated to the wound area, were counted at 48h. The results are expressed as fold compared to HAF1 cell line. D. Phosphorylated and total ERK1/2 protein levels, as well as AKT and pAKT levels were analyzed by western blot in the three cell lines cultured in basal conditions with 10%FBS. Image shown is representative of three independent experiments. Results are means ± S.E.M. of four independent experiments. *P< 0.005 versus HAF1 cells; #P<0.005 versus HCT116 cells. (TIF) [file pone.0130543.s001.tif]

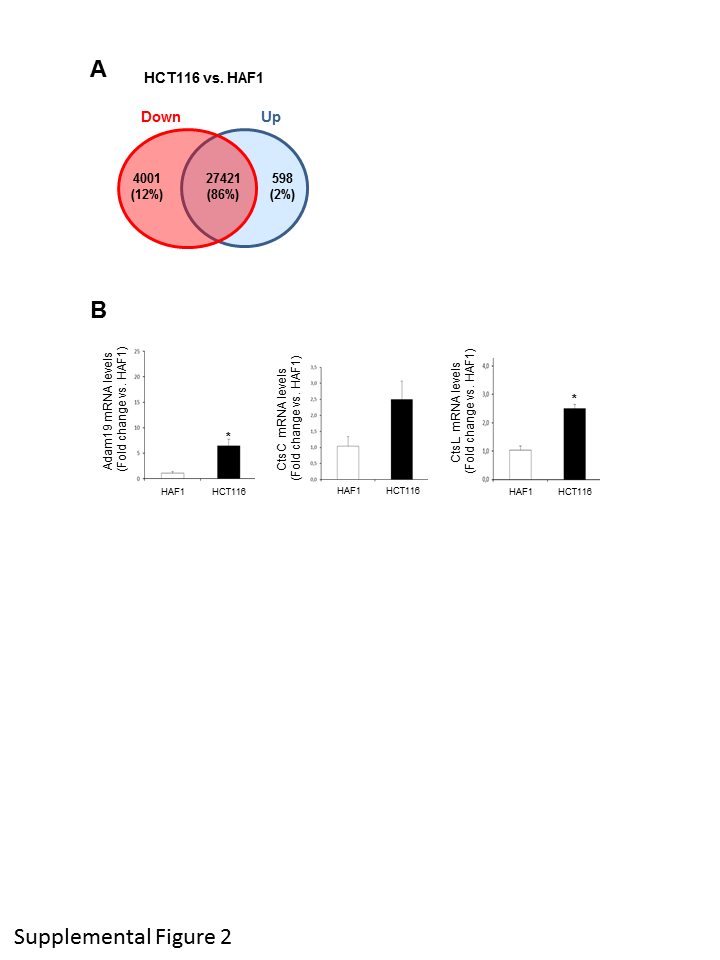

Supplement: S2 Fig — Total RNA from HCT116 CRC cells cultured in basal conditions with 10% FBS was analyzed by microarray. A. Venn-diagram showing the percentage of up- and down-regulated genes found in the parental cell line harboring a KRAS G13D mutation (HCT116), when compared to mRNA levels found in HAF1 cells (KRAS A146T ). B. CATHEPSIN L, CATHEPSIN C and ADAM19 were compared using qPCR. *P< 0.05 versus HAF1 cells. (TIF) [file pone.0130543.s002.tif]

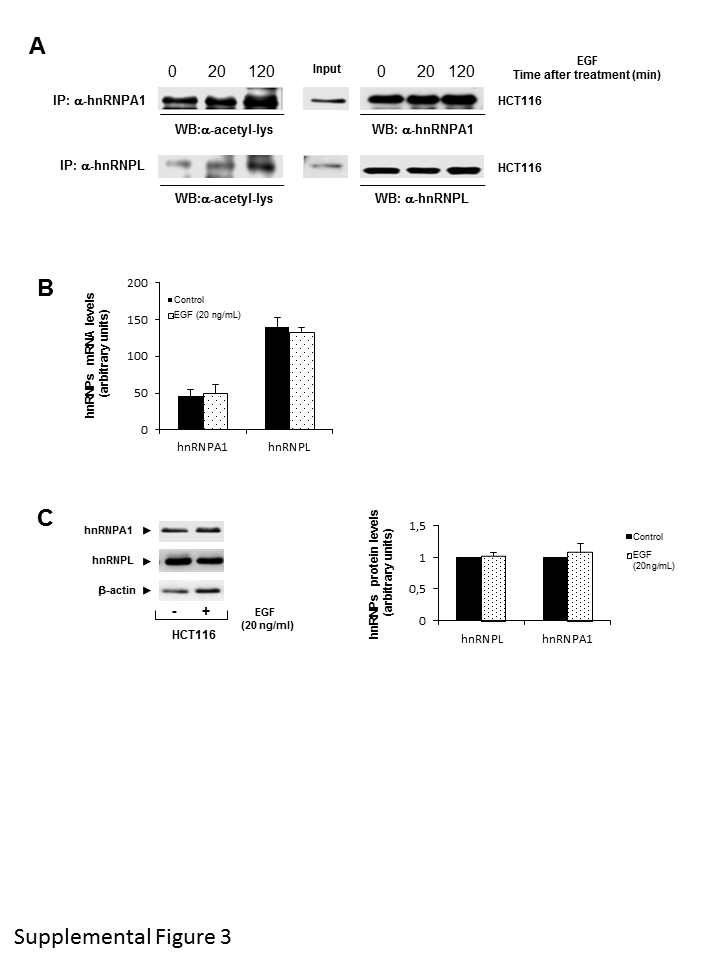

Supplement: S3 Fig — HCT116 cells were grown in serum-free medium and then stimulated with EGF (20ng/mL) for 20 and 120min. A. Protein extracts from control or EGF-treated cells were isolated and immunoprecipitated with α-hnRNPA1 or hnRNPL antibodies. The immunoprecipitated samples were then analyzed by western blot with antibodies recognizing acetyl-lysine residues and, either hnRNPA1 or hnRNPL. Inputs of each specific hnRNP in the different cell lines are shown. B. mRNA levels of hnRNPA1 and hnRNPL were analyzed by qPCR in control and 2h EGF-treated cells. No statistical significance was found (n = 3). C. Western blot analysis showing protein levels of both hnRNPs in control and 2h EGF-treatment conditions. The intensity of hnRNPs bands was measured and normalized by β-actin; graph shows the quantification for both hnRNPL and A1 in HCT116 cell line. No statistical significance was found (n = 3). (TIF) [file pone.0130543.s003.tif]
