# Supplementary material for: EGF-Induced Acetylation of Heterogeneous Nuclear Ribonucleoproteins Is Dependent on KRAS Mutational Status in Colorectal Cancer Cells
Source: PLoS One. 2015 Jun 25;10(6):e0130543. doi: 10.1371/journal.pone.0130543 (PMC4482484; doi:10.1371/journal.pone.0130543)
Supplement: S1 File — (DOCX) [file pone.0130543.s004.docx]

**Supplementary Material and Methods**

**DNA extraction and Somatic mutations sequencing**

For sequencing studies, cells were grown to confluency and DNA was extracted using the method described in Current Protocols in Molecular Biology.

The most frequently genes found to harbor mutations in CRC were sequenced by the use of both, OncoGenBasic S1 & S1.v2 and S2 &S2.v2 kits (Sequencing Multiplex, Genetest). Mutations in different codons for *BRAF, KRAS, NRAS, AKT* and *PI3K* (Supplementary Table I) were analyzed following manufacturer's instructions.

**Primers sequences**

Specific primers for *hnRNP A1* (forward: TAGGCTGGCAGATACGTTCG and reverse: CGGGCTCTTTAGGAGACTCTG), *hnRNPA3* (forward: TTGTGTGGTAATGAGAGACCCC and reverse: CACTACACGCCCATCAACCT), *hnRNPA2/B1* (forward: CAGCGGCAGTTCTCACTACA and reverse: TTTCTCTCTCCATCGCGGAC), *hnRNPL* (forward: CACCCCGCAGAATATGGAGG and reverse: CACTGGTGGACCCATCCTTC) and *GAPDH* (forward: CCAAGGTCATCCATGACAAC and reverse: TGTCATACCAGGAAATGAGC) were designed using the on-line tool Primer-BLAST.

**Cell viability MTT assay**

MTT (3-(4,5-Dimethylthiazol-2-yl)-2,5-diphenyltetrazolium bromide), is reduced to purple formazan in living cells. MTT solution was prepared with 1.0mg of MTT per ml of Mc Coy’s culture media, filtered through 0.2μm filter and protected from light until further use. Culture media was removed and 2 washes with warm (37°C) PBS were performed. Plates were incubated with MTT solution for 1h and washed once with PBS 1x. Then n-propanol was added and the plates were incubated for 30min under mild agitation and protected from light. One ml of the n-propanol was collected onto a microcentrifuge tube and centrifuged for 2min at 12000 g. Finally, absorbance of diluted n-propanol was measured at 560nm.

**Short-term Cell adhesion assay**

Culture flasks (35 mm^2^ and 2x2 mm grid) were coated with fibronectin (2.5 μg/cm^2^) at 37ºC for 1h followed by two washes with PBS. Cells were seeded at a density of 5x10^5^/ plate and incubated at 37^o^C for 2 hours. Non-adherent cells were removed by washing with McCoy’s Medium. Adhered cells were calculated as the average from five grids/plate counted manually under an inverted microscope (Nikon Eclipse Ti).
