# Supplementary material for: EGF-Induced Acetylation of Heterogeneous Nuclear Ribonucleoproteins Is Dependent on KRAS Mutational Status in Colorectal Cancer Cells
Source: PLoS One. 2015 Jun 25;10(6):e0130543. doi: 10.1371/journal.pone.0130543 (PMC4482484; doi:10.1371/journal.pone.0130543)
Supplement: S1 Table — (DOCX) [file pone.0130543.s007.docx]

**Supplementary Table I. Genes and codons sequenced for detection of somatic mutations in CRC cell lines.**

| **Gen** | **Exon** | **Codons** |
| --- | --- | --- |
| *BRAF* | 15 | 472, 594, 596, 600 |
| *KRAS* | 2  3  4 | 12, 13  61  117, 146, 147 |
| *NRAS* | 2  3  4 | 12, 13  61  117, 146, 147 |
| *AKT1* | 3 | 17 |
| *PIK3CA* | 2  4  7  9  20 | 60, 88, 110, 111  345,  420,  542, 545, 546, 549  1025, 1043, 1047, 1049 |
